# Microbial volatile organic compounds: Antifungal mechanisms, applications, and challenges

**DOI:** 10.3389/fmicb.2022.922450

**Published:** 2022-07-15

**Authors:** Xixi Zhao, Jingyi Zhou, Ruofei Tian, Yanlin Liu

**Affiliations:** College of Enology, Northwest A&F University, Yangling, China

**Keywords:** biological control, volatile organic compounds, post-harvest diseases, antifungal mechanism, commercial application

## Abstract

The fungal decay of fresh fruits and vegetables annually generates substantial global economic losses. The utilization of conventional synthetic fungicides is damaging to the environment and human health. Recently, the biological control of post-harvest fruit and vegetable diseases *via* antagonistic microorganisms has become an attractive possible substitution for synthetic fungicides. Numerous studies have confirmed the potential of volatile organic compounds (VOCs) for post-harvest disease management. Moreover, VOC emission is a predominant antifungal mechanism of antagonistic microorganisms. As such, it is of great significance to discuss and explore the antifungal mechanisms of microbial VOCs for commercial application. This review summarizes the main sources of microbial VOCs in the post-harvest treatment and control of fruit and vegetable diseases. Recent advances in the elucidation of antifungal VOC mechanisms are emphasized, and the applications of VOCs produced from antagonistic microorganisms are described. Finally, the current prospects and challenges associated with microbial VOCs are considered.

## Introduction

Fruit and vegetable losses are between 35 and 55% of their production volume, depending on the region. Considering that the decay of fruits and vegetables is a consequence of phytopathogen proliferation on their edible parts, various new anti-phytopathogenic strategies are actively being investigated (Leneveu-Jenvrin et al., 2020). Generally, fruits are protected against decay using chemical substances; however, consumer acceptance of such pesticides is decreasing considering their associated environmental pollution and possible harmful health effects. Furthermore, pathogens can develop resistance against pesticides such as carbendazim and diethofencarb (Ocampo-Suarez et al., 2017). SO_2_ can be used as a fungicide during fruit storage and can inhibit post-harvest pathogen growth in grapes, reduce fruit respiration rate, and maintain fruit quality. However, excess SO_2_ can cause bleaching of grapes, and the SO_2_ residue can be detrimental to human health (Considine and Foyer, 2015). Several areas, such as the United States and the European Union, have promoted the “Integrated Pest Management (IPM)” project to reduce and ultimately eliminate chemical pesticide usage (Diaz et al., 2020). Therefore, it is crucial to develop safer, environmentally friendly, and effective methods against pathogenic fungi.

The usage of antagonistic microorganisms in the biological control of post-harvest fruit and vegetable diseases may be a promising substitute for synthetic fungicides. Antagonistic microorganisms inhibit pathogenic growth *via* competition for nutrients and space, parasitism, antibiosis, host resistance induction, volatile organic compound (VOC) emission, and biofilm formation (Zhang et al., 2020). In particular, VOC emission is a predominant antifungal mechanism of antagonistic microorganisms. Accordingly, Contarino et al. (2019) demonstrated that VOCs produced by *Wickerhamomyces anomalus*, *Metschnikowia pulcherrima*, *Aureobasidium pullulans*, and *Saccharomyces cerevisiae* effectively inhibited post-harvest pathogenic molds. Moreover, each antagonist can produce a wide variety of VOCs. The main VOCs emitted by biological control yeasts are alcohols (ethanol, 3-methylbutan-1-ol, and 2-phenylethanol) and esters (ethyl acetate and 3-methylbutyl acetate). Volatiles do not require antagonistic microorganisms direct contact with food and are, thus, currently considered potential biofumigants.

VOCs derived from antifungal bacteria, filamentous fungi, and yeasts are used for the control of pathogenic fungi in fruits and vegetables. Herein, we review all reported biological control strategies of VOCs for the management of pathogenic fungi in fruits and vegetables. First, microbial-derived VOCs are discussed in detail, whereafter, recent advances in the elucidation of VOC antifungal mechanisms are emphasized. Finally, the applications of VOCs derived from biological control microbes, and the associated prospects and challenges are reviewed.

## VOCs derived from biological control microbes

### Yeast-derived VOCs

**Figure 1:**
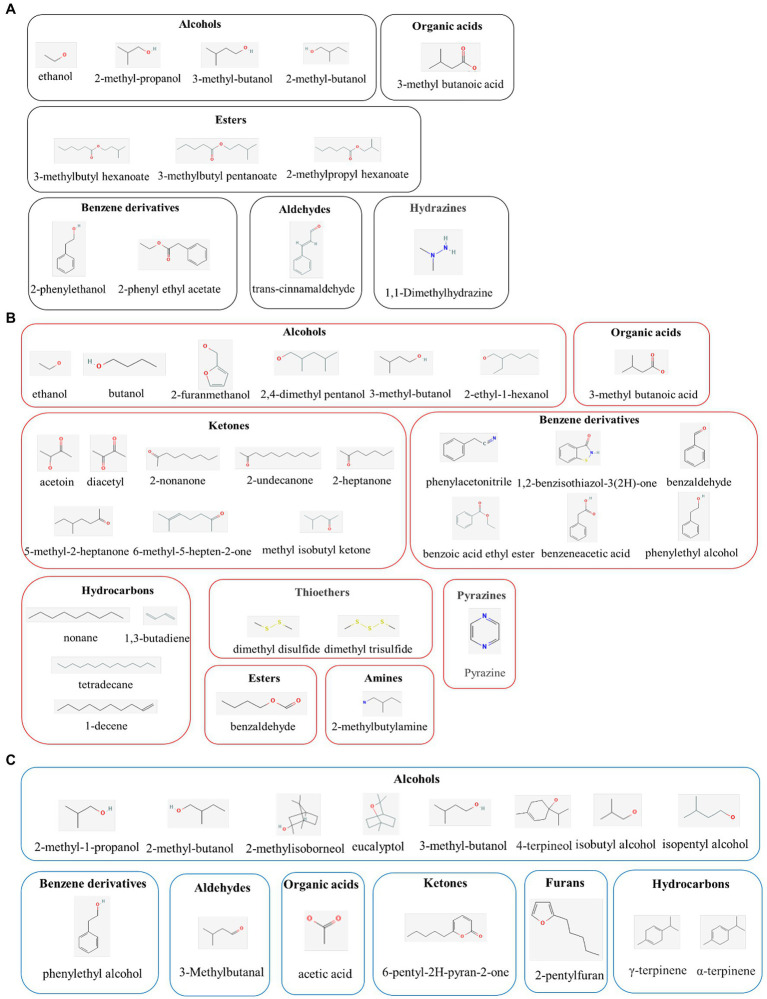
Molecular classes of major active compounds in volatile organic compounds (VOCs) from yeast **(A)**, bacterial **(B)**, and fungal **(C)**. The active compounds in VOCs exert promising antimicrobial activities in the biological control of plant pathogens.

The application of yeast as a biological control agent (BCA) has been extensively studied; seeing as they are environmentally friendly, have no negative toxicological impacts, and their large-scale cultivation is effortless and cost-effective (Mari et al., 2016). Moreover, yeast strains, such as *Aureobasidium* spp., *Candida* spp., *Kloeckera* spp., *Metschnikowia* spp., *Pichia* spp., *Saccharomyces* spp., *Rhodotorula* spp., and *Wickerhamomyces* spp., have been reported to have antifungal properties (Lemos Junior et al., 2020). Various mechanisms have been postulated to describe these antifungal properties, including enhanced natural host defenses, competition for nutrients, and antifungal VOC production. Among these mechanisms, the production of VOCs may be particularly pertinent to the antifungal properties of yeast (Figure 1A; Table 1).

**Table 1:** Main yeasts emitting VOCs, their target pathogen and primary components.

Antagonist	Target	Main VOCs	Reference
*A. pullulans*	*B. cinerea*; *A. alternata*	Ethanol; 2-methyl-propanol; 3-methyl-butanol; 2-phenylethanol	Yalage Don et al., 2020
*W. anomalus* *M. pulcherrima* *A. pullulans* *S. cerevisiae*	*B. cinerea*; *P. digitatum*; *P. italicum*	-	Parafati et al., 2017
*A. pullulans*	*B. cinerea*; *C. acutatum*; *P. expansum*; *P. digitatum*; *P. italicum*	2-phenylethanol	Di Francesco et al., 2015
*H. uvarum*	*B. cinerea*	trans-cinnamaldehyde	Guo et al., 2019
*S. cerevisiae*	*P. guajava*	3-methyl-1-butanol; 2-methyl-1-butanol	Dalilla et al., 2015
*C. jadinii*	*A. carbonarius*	2-phenylethanol	Farbo et al., 2018
*L. thermotolerans* *C. jadinii* *C. friedrichii* *C. intermedia*	*A. ochraceus*	-	Fiori et al., 2014
*S. cerevisiae* *W. anomalus* *M. pulcherrima*	*B. cinerea*	-	Parafati et al., 2015
*C. intermedia*	*A. carbonarius*	2-phenylethanol	Tilocca et al., 2019
*C. sake*	*P. expansum*; *B. cinerea*; *A. alternata*; *A. tenuissima*; *A. arborescens*	3-methylbutyl hexanoate; 3-methylbutyl pentanoate; 2-methylpropyl hexanoate	Arrarte et al., 2017
*K. heveanensis*	*A. flavus*	3-methyl-1-butanol; 2-methyl-1-butanol; 1,1-dimethyl hydrazine; 3-methyl butanoic acid	Jaibangyang et al., 2021
*C. nivariensis*	*A. flavus*	1-pentanol	Jaibangyang et al., 2020
*P. anomala* *P. kluyveri* *H. uvarum*	*A. flavus*	2-phenyl ethyl acetate	Masoud et al., 2005
*D. nepalensis*	*C. gloeosporioides*	phenylethyl alcohol	Zhou et al., 2018

Volatile organic compounds produced by *Pichia* spp. reduce the incidence of *Monascus purpureus* by up to 39.22%, and 2-phenylethanol elicits its antifungal effect on *M. purpureus* by inducing protein synthesis and DNA damage (Zhang et al., 2021). Moreover, 2-phenylethanol is also the main antifungal VOC produced by *Candida intermedia*. VOCs produced by *C. intermedia* can affect protein biosynthesis, proliferative activity, mitochondrial metabolism, and detoxification. Accordingly, *C. intermedia*-produced VOCs and 2-phenylethanol successfully inhibit *Aspergillus carbonarius* radial mycelial growth and reduce ochratoxin A (OTA) production (Tilocca et al., 2019). Furthermore, Ruiz-Moyano et al. (2020) found that VOCs produced by *Hanseniaspora uvarum* effectively controlled the incidence of *Botrytis cinerea* in strawberries and cherries; the main VOCs identified included acetic acid, octanoic acid, ethyl propanoate, N-propyl acetate, 2-methylpropyl acetate, 2-methylbutyl acetate, furan-2-ylmethyl acetate, benzyl acetate, 2-phenylethyl acetate, and heptan-2-one.

Masoud et al. (2005) demonstrated that, during coffee processing, VOCs produced by *Pichia anomala*, *Pichia kluyveri*, and *H. uvarum* inhibited the growth of *Aspergillus ochraceus* and prevented the production of OTA. Moreover, the most effective VOC was 2-phenylethyl acetate, which completely inhibited *A. ochraceus* growth at 48 μg/L headspace. Similarly, VOCs produced by *Saccharomyces* spp. hampered spore production and mycelial growth of *A. carbonarius* and *A. ochraceus*. Among the Culture Collection of Agricultural Microbiology (CCMA) *Saccharomyces* spp. strains, *S. cerevisiae* CCMA 0159, 1,299, and 1,302 exhibited the most efficient *in vitro* inhibition of ochratoxigenic fungi, while also reducing *in vivo* OTA production to 0.04–10.11 μg/kg (de Souza et al., 2021).

Jaibangyang et al. (2020) identified *Candida nivariensis* DMKU-CE18 as an effective VOC-producing (mainly pentan-1-ol) yeast against the growth and conidial germination of *Aspergillus flavus*. Moreover, Jaibangyang et al. (2021) revealed that *Kwoniella heveanensis* DMKU-CE82-produced VOCs induced conidia structural damage, inhibited mycelia and conidiophore development, and reduced aflatoxin B1 (AFB1) production to less than 20 ppb in *A. flavus* contaminated corn grains. The major VOCs produced by *K. heveanensis* DMKU-CE82 were closely matched to 3-methylbutan-1-ol, 2-methylbutan-1-ol, 1,1-dimethylhydrazine, and 3-methylbutanoic acid. *Debaryomyces nepalensis*-produced VOCs predominantly include 2-phenylethanol and can play important roles in the suppression of *Colletotrichum gloeosporioides*. This is of great significance, considering that *C. gloeosporioides*-induced fruit anthracnose results in tremendous economic losses due to the latency of the infection (Zhou et al., 2018).

### Bacteria-derived VOCs

Volatile organic compounds produced by bacteria have low molecular weights and polarities and can effortlessly diffuse through porous soil structures and over great atmospheric distances (Figure 1B; Table 2). These properties significantly contribute to the potential applications of bacterial VOCs in various environments, including in plantation fields and greenhouses, and during storage (Arrarte et al., 2017). Correspondingly, several studies have demonstrated the potential applications of bacterial VOCs in plant disease management and in post-harvest disease control (Dhouib et al., 2019; Calvo et al., 2020).

**Table 2:** Main bacteria emitting VOCs, their target pathogen and primary components.

Antagonist	Target	Main VOCs	Reference
*Bacillus methylotrophicus* *B. thuringiensis*	*Fusarium oxysporum*; *Botryosphaeria* sp.; *Trichoderma atroviride*; *C. gloeosporioides*; *P. expansum*	alcohols; phenols; ketones hydrocarbons; aldehydes esters; acids; pyrazines	He et al., 2020
*Paenibacillus ehimensis*	*C. gloeosporioides*	2-furanmethanol; phenylacetonitrile; 2,4-dimethyl pentanol	Coconubo Guio et al., 2020
*B. velezensis*	*B. cinerea*; *M. fructicola*; *M. laxa*; *P. italicum*; *P. digitatum*; *P. expansum*	2-nonanone; 2-undecanone 2-heptanone; butanol; acetoin; benzaldehyde; butyl formate; diacetyl; nonane; pyrazine	Calvo et al., 2020
*B. subtilis*	*M. fructicola*	-	Zhou et al., 2019
*B. pumilus*	*A. alternata*; *Cladosporium ladosporioides*; *Curvularia lunata*; *F. oxysporum*; *P. italicum*	methyl isobutyl ketone; ethanol; 5-methyl-2-heptanone; 2-methylbutylamine	Morita et al., 2019
*B. amyloliquefaciens* *B. artrophaeus*	*R. solanacearum*	benzaldehyde; 1,2-benzisothiazol-3(2H)-one; 1,3-butadiene	Tahir et al., 2017
*Paenibacillus polymyxa*	*Verticillium longisporum*	2-nonanone; 3-hydroxy-2-butanone	Rybakova et al., 2017
*P. fluorescens*	*P. italicum*	dimethyl disulfide; dimethyl trisulfide	Wang et al., 2021c
*Pseudomonas* sp. (No. 3, No. 35) *Enterobacter* sp. (No. 26, No. 34) *Ralstonia* sp. (No. 50) *Bacillus* sp. (No. 62) *Arthrobacter* sp. (No.146) *Brevibacillus* sp. (No. 2–18) *Paenisporosarcina* sp. (No. 2–60)	*R. solani*	benzoic acid ethyl ester; 3-methylbutanoic acid; 2-ethyl-1-hexanol; 3-methyl-1-butanol; 6-methyl-5-hepten-2-one	Wang et al., 2021a
*B. velezensis*	*V. dahlia*	tetradecane; benzeneacetic acid; benzaldehyde; 1-decene; phenylethyl alcohol	Dhouib et al., 2019
*B. subtilis*	*M. circinelloides*; *F. arcuatisporum*; *A. iridiaustralis*; *C. fioriniae*	2,3-butanedione; 3-methylbutyric acid	Ling et al., 2021

The antifungal capability of *Bacillus subtilis* on post-harvest citrus was first reported in the 1950s (Wilson and Chalutz, 1989). Recently, VOC emissions were identified as the main antifungal mechanism of the *Bacillus* spp. strains. Massawe et al. (2018) identified eight *Bacillus* spp.-produced VOCs that reduced sclerotial production and inhibited mycelial growth of *Sclerotinia sclerotiorum*. Moreover, VOCs emitted by the *B. subtilis* CL2 strain inhibited the hyphal growth of four pathogenic fungi (*Mucor circinelloides* LB1, *Fusarium arcuatisporum* LB5, *Alternaria iridiaustralis* LB7, and *Colletotrichum fioriniae* LB8) and significantly reduced the weight loss rate and decay incidence of wolfberry fruits. The main active antifungal substances in these VOCs are butane-2,3-dione and 3-methylbutanoic acid (Ling et al., 2021). VOCs produced by *Bacillus pumilus* and *Bacillus thuringiensis* significantly inhibit the *in vitro* mycelia growth of *C. gloeosporioides*. Accordingly, the inhibition incidences of inoculated mangos exposed to the VOCs of *B. pumilus* and *B. thuringiensis* were 94.3 and 87.6%, respectively (Zheng et al., 2013). VOCs produced by *Bacillus velezensis* significantly inhibit *in vitro* and fruit borne *B. cinerea*, *Monilinia fructicola*, *Monilinia laxa*, *Penicillium italicum*, *Penicillium digitatum*, and *Penicillium expansum* growth; particularly *M. laxa* (66%), *M. fructicola* (72%), *P. italicum* (80%), and *B. cinerea* (100%). These VOCs mainly include nonan-2-one, undecan-2-one, heptan-2-one, butan-1-ol, 3-hydroxybutan-2-one, benzaldehyde, butyl formate, butane-2,3-dione, nonane, and pyrazine (Calvo et al., 2020). Furthermore, the *B. velezensis*-produced VOCs; tetradecane, 2-phenylacetic acid, benzaldehyde, dec-1-ene, and 2-phenylethanol, also exhibit significant antifungal activity against *Verticillium dahliae*. In addition, *B. velezensis* application significantly reduces the incidence of Verticillium wilt by 70.43 ± 7.08% in tomato plants (Dhouib et al., 2019).

Wang et al. (2020) reported that VOCs produced by the antagonistic bacteria, *Pseudomonas fluorescens* ZX significantly inhibited mycelial growth and conidial germination of *P. italicum* by 42.14 and 77.86%, respectively. Moreover, the primary active antifungal constituents of these *P. fluorescens* ZX-produced VOCs included organic acids and sulfur compounds (Wang et al., 2021c).

The VOCs derived from endophytic bacterial strains also exhibit antifungal activity against pathogens. Accordingly, *Pseudomonas stutzeri* E25 and *Stenotrophomonas maltophilia* CR71 inhibits *B. cinerea* growth *via* VOC emission, with (methyldisulfanyl) methane as the main component (Rojas-Solís et al., 2018). VOCs produced by tomato-derived endophytic bacterial strains, such as *Bacillus nakamurai*, *Bacillus pseudomycoides*, *Bacillus proteolyticus*, *B. thuringiensis*, *Enterobacter asburiae*, and *Enterobacter cloacae*, exhibit antifungal activity against *B. cinerea* (Chaouachi et al., 2021).

Volatile organic compounds produced by *Pseudomonas* sp. (No. 3, No. 35), *Enterobacter* sp. (No. 26, No. 34), *Ralstonia* sp. (No. 50), *Bacillus* sp. (No. 62), *Arthrobacter* sp. (No. 146), *Brevibacillus* sp. (No. 2–18), and *Paenisporosarcina* sp. (No. 2–60) exhibits varying inhibitory effects (7.84–100%) on *Rhizoctonia solani* growth. In particular, *Ralstonia* sp. completely inhibits the growth of *R. solani* as a result of VOC production, among which ethyl benzoate, 3-methylbutanoic acid, 2-ethylhexan-1-ol, 3-methylbutan-1-ol, and 6-methylhept-5-en-2-one are confirmed to be toxic to *R. solani* (Wang et al., 2021a). Additionally, *R. solani* is also inhibited by VOCs derived from several *Streptomyces* spp. soil isolates, in which the effective VOC constituents include methyl 2-methylpentanoate and 1,3,5-trichloro-2-methoxy benzene (Cordovez et al., 2015).

Gómez et al. (2021) identified several anti-phytopathogenic marine actinobacteria, including *Streptomyces* sp. (PNM-149), which exhibited antifungal activity against *C. gloeosporioides* growth *via* two major VOC components (methyl 2-aminobenzoate and 1,2,7,7-tetramethylbicyclo[2.2.1]heptan-2-ol). Moreover, *Bacillus atrophaeus* elicits significant inhibition against various fungal pathogens. The primary inhibitory VOCs produced by *B. atrophaeus* HAB-5 against *C. gloeosporioides* included 2-chloroacetic acid, tetradecyl esters, octadecane, and methyl hexadecanoate (Rajaofera et al., 2019). Overall, these studies provide a foundation for the application of antagonistic bacteria in the control of fungal infections.

### Fungi-derived VOCs

In addition to yeast and bacteria, several fungal species elicit biological control activities by producing VOCs (Figure 1C; Table 3). Among the antagonistic fungi, the yeast-like fungus *A. pullulans* is recognized as a propitious post-harvest disease BCA (Di Francesco et al., 2020a). Accordingly, *Alternaria alternata* and *B. cinerea* conidia germination and colony growth are suppressed by *A. pullulans*-derived VOCs, including ethanol and 2-phenylethanol as the key inducers of this inhibitory effect (Di Francesco et al., 2015). Moreover, VOCs derived from *A. pullulans* L1 and L8 inhibit *Monilinia fructigena* mycelium growth (70 and 50%, respectively) and *M. fructicola* conidia germination (85 and 70%, respectively). As the most active compound among the VOCs, 2-phenylethanol displays inhibitory action against all the pathogens on cherry and apricot fruits (Di Francesco et al., 2020b). In addition, VOCs generated by *Aureobasidium subglaciale*, of which 3-methylbutan-1-ol is the most effective, inhibit *B. cinerea* mycelial growth (65.4%; Di Francesco et al., 2020a). VOCs produced by *A. pullulans*, which mainly include ethanol, 2-methylpropan-1-ol, 3-methylbutan-1-ol, and 2-phenylethanol, can increase intracellular reactive oxygen species (ROS) accumulation, lipid peroxidation, and content, leakage, thereby inhibiting *B. cinerea* growth (Don et al., 2020).

**Table 3:** Main fungus emitting VOCs, their target pathogen and primary components.

Antagonist	Target	Main VOCs	Reference
*Trichoderma asperelloides*	*Colletotrichum* sp.; *C. cassiicola*; *C. lunata*; *Ganoderma* sp.; *P. oxalicum*; *N. clavispora*; *S. rolfsii*; *S. cucurbitacearum*	2-methyl-1-butanol; 2-pentylfuran; acetic acid; 6-pentyl-2H-pyran-2-one	Phoka et al., 2020
*Diaporthe apiculatum*	*A. alternata*; *Botryosphaeria dothidea*; *B. cinerea*; *Cercospora asparagi*; *C. gloeosporioides*; *Fusarium graminearum*; *Sphaeropsis sapinea*; *Valsa sordida*	γ-terpinene; α-terpinene; 4-terpineol	Song et al., 2019
*Hypoxylon anthochroum*	*F. oxysporum*	eucalyptol	Macias-Rubalcava et al., 2018
*Streptomyces alboflavus*	*Fusarium moniliforme*; *A. flavus*; *A. ochraceus*; *Aspergillus niger*; *Penicillum citrinum*	2-methylisoborneol	Wang et al., 2013
*A. subglaciale*	*B. cinerea*	3-methyl-1-butanol	Di Francesco et al., 2020b
*A. pullulans*	*M. fructigena*; *M. fructicola*	2-phenylethanol	Di Francesco et al., 2020a
*S. globisporus*	*B. cinerea*; *S. sclerotiorum*	-	Li et al., 2012
*A. pullulans*	*B. cinerea*; *C. acutatum*; *P. expansum*; *P. digitatum*; *P. italicum*	2-phenylethanol; 3-methyl-1-butanol; 2-methyl-1-butanol; 2-methyl-1-propanol	Di Francesco et al., 2015
*Trichoderma viride*	*Arabidopsis thaliana*	isobutyl alcohol; isopentyl alcohol; 3-Methylbutanal	Hung et al., 2013
*T. spirale*	*C. cassiicola*; *C. aeria*	6-pentyl-2H-pyran-2-one	Baiyee et al., 2019
*T. spirale*	*F. incarnatum*	phenylethyl alcohol	Intana et al., 2021

*Trichoderma* spp. plays an important role as a BCA in a wide variety of plants (Sunpapao et al., 2018; Baiyee et al., 2019). Accordingly, the major *Trichoderma spirale* T76-1-produced VOC (6-pentylpyran-2-one) suppresses *Corynespora cassiicola* and *Curvularia aeria* growth by 41.29 and 42.35%, respectively (Baiyee et al., 2019). Moreover, *Trichoderma asperellum* T76-14-emitted VOCs, particularly 2-phenylethanol, effectively inhibits *Fusarium incarnatum* growth (62.5%) and rot after 7 days of incubation (Intana et al., 2021).

Li et al. (2010) reported suppressed *P. italicum* spore germination, mycelial growth, sporulation, and disease incidence in inoculated citrus in the presence of *Streptomyces globisporus* JK-1-derived VOCs. Moreover, these VOCs can inhibit *B. cinerea* growth on media and in inoculated tomatoes (Li et al., 2012).

## Antifungal mechanism of VOCs

**Figure 2:**
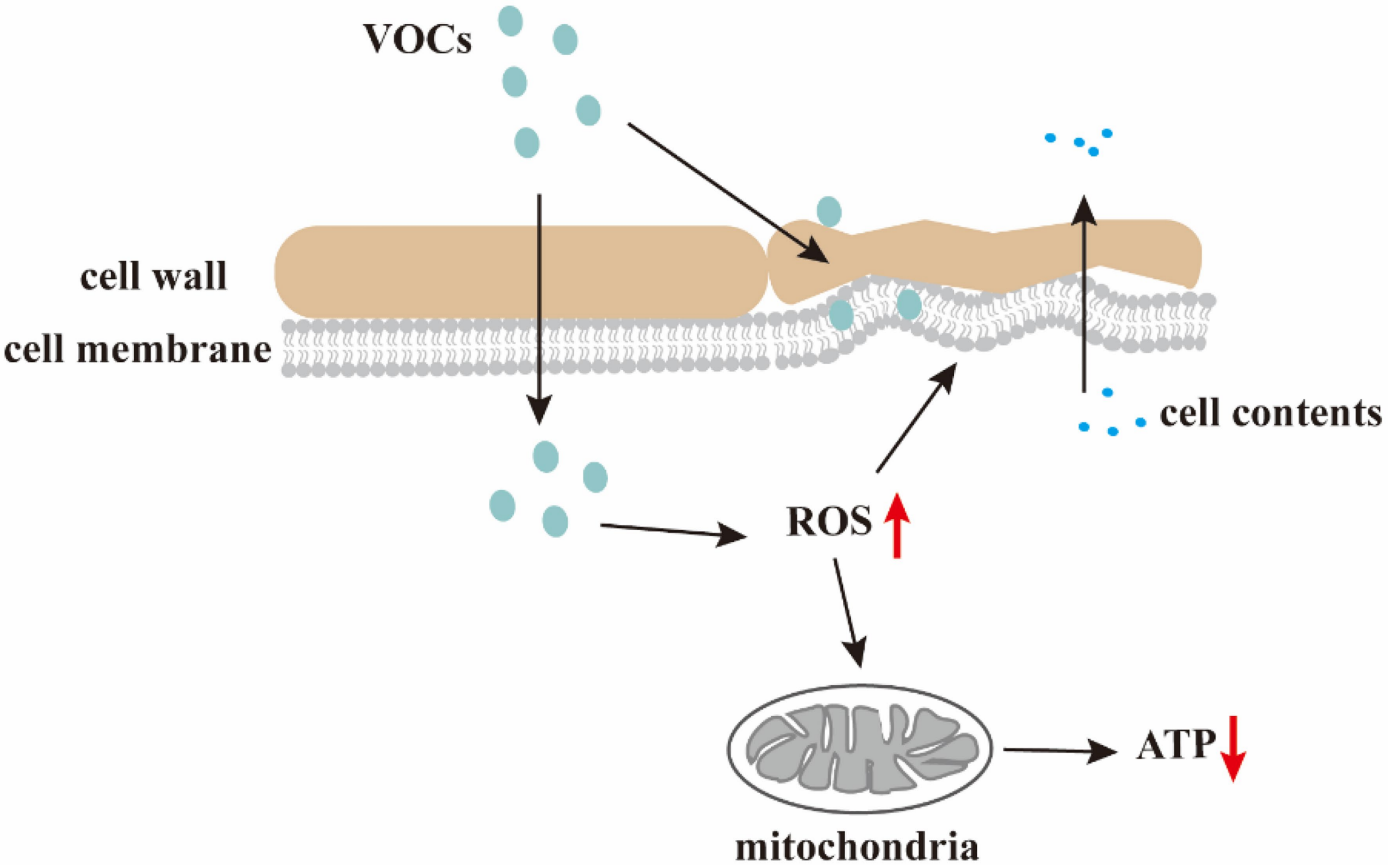
Antifungal mechanism of the VOCs. VOCs damage cell walls and membranes, resulting in changes in the morphology of microbial cells and leakage of cell contents; VOCs disrupt redox balance and increase intracellular ROS level causing membrane lipid peroxidation, mitochondrial dysfunction, and decreased ATP levels.

Limited information exists regarding the molecular and physiological mechanisms by which VOCs control post-harvest diseases. Nevertheless, the main mechanism underlying the antifungal effects of VOCs is the disruption of cell wall and membrane structures, leading to intracellular lysate leakage and oxidative stress induction (Figure 2).

### Disruption of pathogenic fungi cell wall and membrane structures

The shape of microbial cells is maintained by the increased mechanical resistance provided by the cell wall and membrane. The cell wall is composed of three major macromolecules (mannoproteins, β-glucan, and chitin) essential for cell morphology sustainability and protection against mechanical damage. The integrity of fungal cell membranes, which are composed of proteins, phospholipids, and sugars, are vital to the survival of fungi. However, microbial VOCs can damage cell walls and membranes, resulting in microbial morphological changes.

Accordingly, *B. cinerea* treated with *S. globisporus* JK-1-derived VOCs exhibit excessive vesiculation, thickened walls, and retracted membranes (Li et al., 2012). Moreover, *Trichoderma* sp., *Phoma* sp., and *Colletotrichum* sp. exposed to *Chromobacterium vaccinii*-derived VOCs exhibit extensive morphological abnormalities, such as swollen hyphal cells, vacuolar depositions, and cell wall alterations (Ebadzadsahrai et al., 2020). Tahir et al. (2017) reported that benzaldehyde, 1,2-benzothiazol-3-one, and buta-1,3-diene released by *Bacillus* spp. caused morphological and ultra-structural changes in *Ralstonia solanacearum* cells. Correspondingly, Wang et al. (2021b) demonstrated inhibition of *C. gloeosporioides* growth *via B. subtilis CF*-3 VOC-induced downregulation of gene expression related to cell membrane fluidity, wall integrity, energy metabolism, and the production of cell wall-degrading enzymes. In addition, 2,4-ditert-butylphenol, which is a characteristic VOC of *B.subtilis CF*-3, elicits similar inhibitory effects on *C. gloeosporioides*.

Some VOCs directly target fungal cell membranes by increasing membrane permeability and cellular leakage. VOCs, such as organic acids, increase cell membrane fluidity, leading to membrane protein conformational changes, intracellular content leakage, and subsequent fungal cell death. Moreover, the direct insertion of *Pseudomonas* spp.-produced cis-9-heptadecenoic acid [(Z)-heptadec-9-enoic-acid] in the phospholipid bilayer of cell membranes, and subsequent interaction with fungal cell membranes increases membrane fluidity and eventuates in the death of pathogenic fungi such as *B. cinerea* (Avis and Belanger, 2001). Furthermore, Bergsson et al. (2001) demonstrated that decanoic acid destroyed *Candida albicans* cell membranes, resulting in the outflow of cytoplasmic contents and rapid, effective elimination of the pathogen fungi.

Volatile organic compounds also alter fungal membrane permeability *via* peroxidation of membrane lipids. Accordingly, *A. pullulans* VOCs may trigger lipid peroxidation and electrolyte leakage in *B. cinerea* and *A. alternata* (Yalage Don et al., 2021). Additionally, *Psidium guajava* exposure to *S. cerevisiae* VOCs increased the membrane lipid peroxidation plasma membrane permeability (Dalilla et al., 2015). Excessive ROS production alters lipid layer composition and triggers lipid peroxidation *via* the conversion of unsaturated lipids to polar lipid hydroperoxides (Vazquez et al., 2019). Moreover, extensive lipid peroxidation-induced alterations in cell membrane permeability result in membrane disintegration, free radical chain reactions, and eventual cell death (Massawe et al., 2018).

In summary, cell and organelle membranes are potential VOC targets *via* membrane damage-induced cell structure deformation and cytoplasmic inclusion of organelle material. Moreover, VOCs may enter fungal cells *via* hydrogen bonding. Consequently, the forces created during this bonding disturb the aqueous solution of cell membranes and interfere with cellular physiology and functionality.

### Effects of oxidative stress on fungal cells

Volatile organic compounds derived from biological control microbes trigger ROS accumulation and oxidative stress in fungal cells. Excessive ROS accumulation disrupts the redox balance, reacts with cellular macromolecules, such as lipids, proteins, and DNA, and eventuates in cell dysfunction or death.

Massawe et al. (2018) identified four endophytic *Bacillus* spp. VOCs that strongly induced ROS production in *S. sclerotiorum* mycelial cells. Insufficient detoxification of ROS by cellular antioxidant defense mechanisms, such as catalase (CAT) and superoxide dismutase (SOD) activities, results in oxidative stress. As such, the increased CAT and SOD activity observed by Fialho et al. (2014) in *Guignardia citricarpa* mycelia following *S. cerevisiae* VOC exposure indicated VOC-induced imbalanced fungal redox states.

Fialho et al. (2014) reported that *S. cerevisiae* CR-1 VOCs inhibited *G. citricarpa* growth by disrupting the intracellular redox homeostasis and triggering harmful ROS accumulation. Moreover, Xie et al. (2020) reported that the *B. subtilis* DZSY21 VOC, 3-methylbutyl acetate, strongly induced intracellular ROS accumulation and inhibited mycelia growth and conidial sporulation of *Curvularia lunata*. Ye et al. (2020) observed significant inhibition of *Fusarium oxysporum* f. sp. *cucumerinum* by *Corallococcus* sp. EGB VOCs, particularly 6-methylheptan-1-ol. Accordingly, ROS accumulation and gradual fungal cell apoptosis occurred following 6-methylheptan-1-ol treatment.

Reactive oxygen species are mainly generated during aerobic respiration *via* the complex I enzyme of the mitochondrial respiratory chain (Yalage Don et al., 2021). Furthermore, Zhang et al. (2021) demonstrated hypha morphological changes, cell membrane destruction, ergosterol reduction, and significant ROS accumulation in *Ceratocystis fimbriata* cells following exposure to *Pseudomonas chlororaphis* subsp. aureofaciens SPS-41 VOCs. Consequentially, oxidative stress-induced mitochondrial dysfunction and decreased ATP levels inhibited the growth of *C. fimbriata*. Moreover, *S. cerevisiae*-produced decanoic acid significantly decreases intracellular ATP levels and inhibits *B. cinerea* growth, possibly *via* mechanisms related to energy metabolism. Stevens and Hofmeyr (1993) demonstrated the cytoplasmic entry of octanoic acid and decanoic acid through *S. cerevisiae* membranes and subsequent H^+^ dissociation, significant cytoplasmic pH decrease, and membrane H^+^-ATPase activation. Cellular ATP exhaustion occurs during H^+^ emission, resulting in growth inhibition. Overall, VOC-induced ROS accumulation and oxidative stress lead to pathogenic fungal growth inhibition; however, the complete mechanism of ROS remains to be further elucidated.

## Biological control applications of microbial-derived VOCs

**Figure 3:**
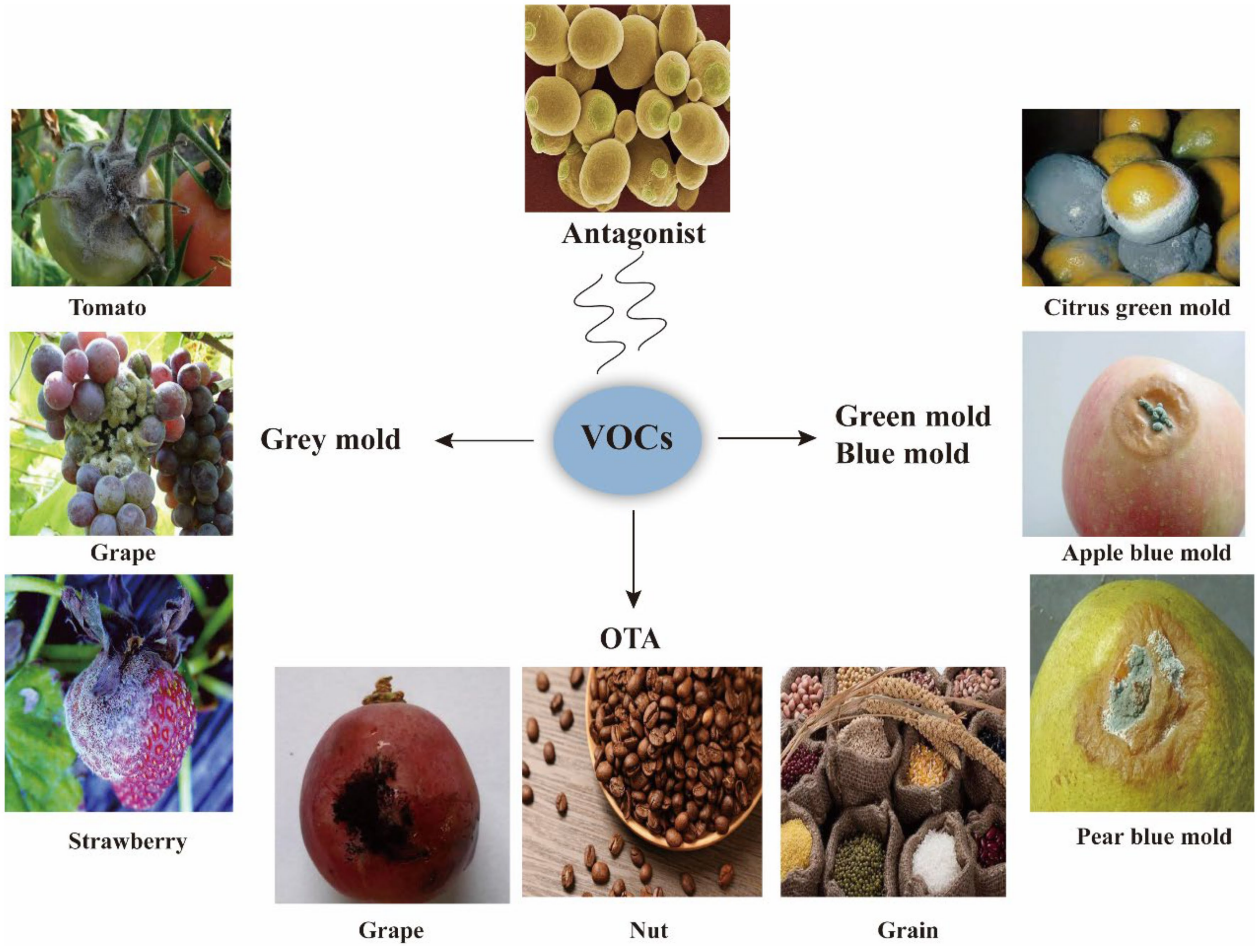
Applications of VOCs. VOCs are applied to inhibit phytopathogenic fungi, such as grey mold, green mold, and blue mold that cause rot in fruit and vegetables; VOCs are also applied to inhibit pathogenic fungi that produce toxins such as OTA.

Microbial VOCs have been applied in the control of diseases, such as grey mold, green mold, and blue mold, and to reduce toxins such as OTA (Figure 3).

Grey mold, caused by *B. cinerea*, is a profound pre- and post-harvest fruit and vegetable disease. More than 200 species of plants, including cucumbers, table grapes, tomatoes, and strawberries, are susceptible to gray mold infection (Huang et al., 2012). Chemical fungicides are utilized in the management of this disease; however, *B. cinerea* effortlessly develops fungicide resistance due to its high genetic variability, prolific reproduction, and short life cycle (Chaouachi et al., 2021). Recently, research has been aimed at the development of environmentally-friendly control methods against *B. cinerea*, including BCA utilization. *Botrytis cinerea* disease incidence and severity on wound-inoculated tomato fruit are inhibited when fumigated with *S. globisporus* JK-1 (Li et al., 2012). Moreover, the bio-fumigation of apples with *A. pullulans* L1 and L8 VOCs effectively controlled *B. cinerea*, *Colletotrichum acutatum*, *P. expansum*, *P. digitatum*, and *P. italicum*. The lesion diameter of apples inoculated with *B. cinerea* following *A. pullulans* L1 and L8 VOC exposure reduced by 88.9 and 94.4%, respectively. Moreover, *A. pullulans* L1 and L8 VOCs reduce the incidence of blue mold and bitter pit on apples by 73.9 and 44.4%, respectively (Di Francesco et al., 2015). The VOCs produced by *M. pulcherrima*, *W. anomalus*, *A. pullulans*, and *S. cerevisiae* are highly effective in controlling gray mold-induced decay of grape berries (Parafati et al., 2015). Huang et al. (2011) demonstrated that *C. intermedia* VOC (cyclooctatetraene and 3-methylbutan-1-ol) exposure significantly reduced the incidence and severity of *Botrytis* spp. strawberry rot. Moreover, the VOCs derived from *Sporidiobolus pararoseus* effectively suppressed strawberry gray mold disease under air-tight conditions (Huang et al., 2012). Similarly, the tomato-derived endophytic *Enterobacter* sp. TR1 VOC (3-methylbutan-1-ol) completely suppresses *B. cinerea* infection and growth at 0.442 ml/L, whereas *Bacillus* spp. protects against fungal infection when applied to vegetative cells of tomatoes. VOCs derived from *B. velezensis* I3 reduce grey mold in grapes by 50%, while those of *B. velezensis* BUZ-14 decrease brown rot severity in apricots (Chaouachi et al., 2021).

The microbial metabolite, OTA, consists of the amino acid, phenylalanine, linked by an amide bond to a pentaketide dihydroisocoumarin. OTA is the second most predominant mycotoxin found in food and feed products, and is classified as a group 2B carcinogen by the World Health Organization (Farbo et al., 2018). Furthermore, OTAs are predominantly produced by *Aspergillus* spp. and *Penicillium* spp. in warm and tropical regions. *Candida intermedia* 235 and *Lachancea thermotolerans* 751 significantly inhibit *A. carbonarius* on grape berries and *in vitro*, while VOCs produced by non-fermenting (*Cyberlindnera jadinii* 273 and *Candida friedrichii* 778) and low-fermenting (*C. intermedia* 235 and *L. thermotolerans* 751) yeast strains may prevent *in vitro A. carbonarius* sporulation. Moreover, *C. intermedia* 235, *L. thermotolerans* 751, and *C. friedrichii* 778 efficiently adsorb artificially spiked OTA from grape juice (Fiori et al., 2014). However, the main antifungal VOC in *C. intermedia* 253 (2-phenylethanol) only partially mimics the metabolic effects of whole yeast VOCs (Tilocca et al., 2019). Additionally, *Saccharomyces* spp.-produced VOCs hamper *A. carbonarius* CCDCA 10608 and *A. ochraceus* CCDCA 10612 spore production and mycelial growth (de Souza et al., 2021).

*Penicillium digitatum* (green mold) and *P. italicum Wehmer* (blue mold) result in significant post-harvest economical losses (Papoutsis et al., 2019). Both *P. digitatum* and *P. italicum* are wound pathogens that produce a large amount of airborne spores (Kellerman et al., 2016). The disease incidence of *P. expansum* on apples in the presence of *Candida sake* VOCs [mainly 3-methylbutyl hexanoate, 3-methylbutyl pentanoate, 2-methylpropyl hexanoate, and ethyl 4-(4-nitrophenyl)-1,3-thiazole-2-carboxylate] is reduced by 53% and the severity by 20%, indicating that *C. sake* VOCs are effective *P. expansum* BCAs in apples. VOCs produced by *W. anomalus* efficiently reduce *P. digitatum* infections in mandarin fruits (Parafati et al., 2017). Moreover, Li et al. (2010) reported that blue mold sporulation and disease incidence on citrus were reduced in the presence of the VOCs from *S. globisporus* JK-1. Accordingly, Wang et al. (2020) reported suppressed citrus blue mold disease incidence and lesion size by VOCs from *P. fluorescens*. Moreover, the *P. fluorescens* VOCs (100 μl/L dimethyl disulfide and 10 μl/L dimethyl trisulfide) completely inhibited blue mold on citrus fruits (Wang et al., 2021c).

## Prospects and challenges

In the post-harvest stage, VOCs may be regarded as ideal BCAs, considering that their activity does not require direct contact with the pathogen or food. However, to effectively apply these BCAs, their underlying antagonistic and pathogenic mechanisms must first be elucidated, thereby allowing for an understanding of their interactions and biology (Calvo et al., 2020). In the pre-harvest stage, VOCs are mainly used in open-field agricultural and horticultural practices. The major challenge for large-scale VOC application in agricultural and horticultural practices is its volatility (Tilocca et al., 2020). Technical progress from controlled conditions to agricultural systems is required to overcome the current scaling limitations of VOC implementation, thereby formulating more effective and productive applications in the field and during post-harvest management.

To date, a large body of research exists on the utilization of VOCs as pre- and post-harvest BCAs. However, limited studies have reached the commercial development stage and launched commercial products. The main reason for this is the general lack of knowledge associated with VOC mechanisms of action. Moreover, the low solubility of VOCs in water limits its aquatic applications (Kanchiswamy et al., 2015).

Toxicity studies are a requisite for all novel BCAs that reach the market (Ocampo-Suarez et al., 2017). Considering that VOC activity ranges from proximal interactions *via* water diffusion to distant interactions *via* air diffusion, the possible hazards of VOCs need to be thoroughly evaluated (Spadaro and Droby, 2016). Some BCAs, such as *Pichia kudriavzevii*, are known nosocomial pathogens and may cause neonatal deaths (Nagarathnamma et al., 2017). Thus, although numerous studies may be generating valuable information in terms of disease control, the associated data would not be suitable for the practical development of BCAs.

## Conclusion

Fruit and vegetable decay results in immense global economic losses and is harmful to human health. Biological control of post-harvest fruit and vegetable diseases by antagonistic microorganisms has been extensively studied, and the post-harvest disease management potential of VOCs has been confirmed; however, successful commercial application of VOCs is yet to be achieved. Therefore, future studies are required to comprehensively elucidate the antifungal mechanisms of VOCs to accommodate the development of antagonistic microorganisms suitable for commercial applications.

## Author contributions

XZ: conceptualization, software, visualization, writing—original draft, and writing—review and editing. JZ: investigation, visualization, software, and writing—review and editing. RT: visualization and writing—review and editing. YL: funding acquisition, project administration, and writing—review and editing. All authors contributed to the article and approved the submitted version.

## Funding

This work was supported by Research start-up funds of Northwest A&F University (Z1090121025), Ningxia Helan Mountain’s East Foothill Wine Experiment and Demonstration Station of Northwest A&F University (750104), and Ningxia Key Research and Development Project (2020BCF01003).

## Conflict of interest

The authors declare that the research was conducted in the absence of any commercial or financial relationships that could be construed as a potential conflict of interest.

## Publisher’s note

All claims expressed in this article are solely those of the authors and do not necessarily represent those of their affiliated organizations, or those of the publisher, the editors and the reviewers. Any product that may be evaluated in this article, or claim that may be made by its manufacturer, is not guaranteed or endorsed by the publisher.
